# Retrospective Study on the Influencing Factors and Prediction of Hospitalization Expenses for Chronic Renal Failure in China Based on Random Forest and LASSO Regression

**DOI:** 10.3389/fpubh.2021.678276

**Published:** 2021-06-15

**Authors:** Pingping Dai, Weifu Chang, Zirui Xin, Haiwei Cheng, Wei Ouyang, Aijing Luo

**Affiliations:** ^1^Key Laboratory of Medical Information Research, Third Xiangya Hospital, Central South University, Changsha, China; ^2^Department of Medical Information, School of Life Science, Central South University, Changsha, China; ^3^Department of Sociology, Central South University, Changsha, China; ^4^Second Xiangya Hospital, Central South University, Changsha, China

**Keywords:** random forest, LASSO regression, chronic renal failure, hospitalization costs, influencing factors, prediction

## Abstract

**Aim:** With the improvement in people's living standards, the incidence of chronic renal failure (CRF) is increasing annually. The increase in the number of patients with CRF has significantly increased pressure on China's medical budget. Predicting hospitalization expenses for CRF can provide guidance for effective allocation and control of medical costs. The purpose of this study was to use the random forest (RF) method and least absolute shrinkage and selection operator (LASSO) regression to predict personal hospitalization expenses of hospitalized patients with CRF and to evaluate related influencing factors.

**Methods:** The data set was collected from the first page of data of the medical records of three tertiary first-class hospitals for the whole year of 2016. Factors influencing hospitalization expenses for CRF were analyzed. Random forest and least absolute shrinkage and selection operator regression models were used to establish a prediction model for the hospitalization expenses of patients with CRF, and comparisons and evaluations were carried out.

**Results:** For CRF inpatients, statistically significant differences in hospitalization expenses were found for major procedures, medical payment method, hospitalization frequency, length of stay, number of other diagnoses, and number of procedures. The *R*^2^ of LASSO regression model and RF regression model are 0.6992 and 0.7946, respectively. The mean absolute error (MAE) and root mean square error (RMSE) of the LASSO regression model were 0.0268 and 0.043, respectively, and the MAE and RMSE of the RF prediction model were 0.0171 and 0.0355, respectively. In the RF model, and the weight of length of stay was the highest (0.730).

**Conclusions:** The hospitalization expenses of patients with CRF are most affected by length of stay. The RF prediction model is superior to the LASSO regression model and can be used to predict the hospitalization expenses of patients with CRF. Health administration departments may consider formulating accurate individualized hospitalization expense reimbursement mechanisms accordingly.

## Introduction

Chronic renal failure (CRF) refers to chronic progressive renal parenchyma damage caused by various factors, leading to obvious kidney atrophy and the inability to maintain basic function. Chronic renal failure is a clinical syndrome characterized by retention of metabolites and water, electrolyte and acid-base disorders, and major clinical manifestations of other organ system involvement. Chronic renal failure has become a major public health problem worldwide. Chronic renal failure can occur at all ages, and there are many differences in the affected population. With the improvement in people's living standards, the CRF incidence is increasing annually, and CRF has become one of the major chronic diseases affecting the health of the Chinese people. A national epidemiological survey conducted by Zhang Lixin et al. (1) in 2012 showed that the overall prevalence of chronic kidney disease in China was 10.8%. According to the annual report of the United States Renal Disease Data System (USRDS) 2016, the global average prevalence of adult CKD is 14.8% (2). A study by the Korean Society of Nephrology (3) also showed that the incidence of end-stage renal disease in South Korea is 70% of that in the United States. The number of patients has increased year by year, and the treatment costs have also increased accordingly. Therefore, the increase in the number of CRF patients has significantly increased pressure on national medical budgets (4).

Mohnen et al. analyzed the medical expenses of patients using different kidney replacement methods based on Dutch health insurance claims data. The results showed that the costs of all dialysis methods are very high, with annual expenditures of 77,566 euros and 92,616 euros for continuous outpatient peritoneal dialysis (CAPD) and central hemodialysis (CHD), respectively, and 105,833 euros for patients in the mixed dialysis group. Most of the overall health care costs are related to renal replacement therapy (RRT) (5). Research by Makhele et al. (6) showed that in South Africa, from the perspective of healthcare providers, the annual cost of hemodialysis (HD) per patient (31,993.12 US dollars) is higher than that of peritoneal dialysis (PD) (25,282.00 US dollars). The treatment for CRF includes conservative medical treatment and surgical treatment, such as continuous PD, HD, and kidney transplantation. The cost of treatment between the HD and PD varies substantially. The hospitalization costs of CRF patients with different comorbidities and accompanying diseases are also different (7–10). A study by Khan of Tufts University School of Medicine in Boston (11) found that secondary hyperparathyroidism is associated with the high cost of treatment for CRF patients with cardiovascular complications. The research of Zhao et al. (12) indicates that the scope of medical insurance payments will affect hospitalization expenses. Different medical insurance reimbursement payment systems may affect the choice of end-stage renal disease treatment, thereby affecting the allocation of related resources and ultimately affecting the national medical budget (13, 14). Therefore, many factors affect the hospitalization costs of CRF patients, and patient grouping and medical insurance payment standards are more complicated, thus necessitating further research (15). Incorporating these potential factors to predict the medical expenditures of hospitalized CRF patients will be beneficial to the development of policies regarding the allocation of health resources.

Data mining is a process involving careful analysis of large amounts of data to reveal meaningful new relationships, trends, and patterns. Data mining emerged in the late 1980s and represents a new field with important application value in database research, which is an intersecting field. The discipline integrates theories and technologies in many fields such as artificial intelligence, database technology, pattern recognition, machine learning, statistics, and data visualization (16, 17). With the development of data mining research and applications, people have reached a consensus on the understanding of data mining; that is, data mining is a method that uses various strategies to extract hidden and potential information and knowledge from a large amount of data, which is very useful for the decision-making process (18). Therefore, data mining provides a new and promising method for reasonable allocation and control of hospitalization expenses, especially in the era of big data (19). Scholars have applied data mining algorithms to predict and analyze medical expenses; Yang et al. (20) used four machine learning models for patients with high-cost and high-demand chronic diseases, including ordinary least squares linear regression (LR), regularized regression (LASSO), gradient boosting machine (GBM), and recurrent neural networks (RNN, a deep learning approach), and constructed a medical expenditure prediction model. Cao et al. (21) proposed the alpha(tj) algorithm and the truncated Newton algorithm to build a dynamic medical path Net system to predict the medical expenses of gastric cancer patients. Wang et al. (22) used the random forest (RF) model to predict the medical expenses of individual diabetic patients and evaluated related influencing factors, but no studies on the prediction of hospitalization expenses and influencing factors for patients with CRF have been performed.

China is currently enacting new medical reform policies, and the medical insurance payment methods advocated by the National Medical Security Administration mainly include diagnosis-related groups (DRGs) and Diagnosis Intervention Packets (DIPs). As a payment tool that can effectively control increase in medical expenses (23), DRGs were conceived in the United States and rapidly developed worldwide. Diagnosis-related groups are based on patient age, sex, the length of hospital stay, factors such as clinical diagnosis, disease, surgery, disease severity, comorbidities, and complications, and outcomes, which divide patients into 500–600 DRGs, and then the amount of compensation that should be given to a hospital is determined. Diagnosis intervention packets are based on the three core elements of disease screening, measuring the score for each disease, and determining the coefficients of medical institutions to establish a disease score database reflecting differences between different diseases. The relative weight of a medical institution establishes the relationship between the cost of diagnosis and treatment of a disease and the payment price, which is the payment method used in China (24). In addition to these two mainstream payment methods, traditional payment methods such as project-based payment are available. These medical expense payment policies are subject to difficulties and deficiencies in the actual implementation of human resources, information technology, and economic development. Thus, data mining and machine learning algorithms are required for innovative integration of various characteristics of diseases according to the currently implemented medical insurance payment methods to explore medical expense payment methods that are more suitable for China's national conditions. Therefore, this study selected CRF inpatients from three tertiary first-class hospitals in Beijing as the research objects. Our purpose is to use the RF method and the LASSO method to predict individual hospitalization expenses and evaluate related factors based on data from the first page of CRF patients' hospital records. From a personal perspective, predicting the cost of CRF hospitalization will render resource allocation more accurate and reasonable. Our research can provide new ideas for health policy and management research.

## Materials and Methods

### Source of Data

In China, national regulations require hospitals at or above the county level to use the International Classification of Diseases (ICD) on the front pages of medical records to classify and code disease diagnoses. In the ICD-10, the three-digit code N18 represents the category of CRF. Therefore, the N18 category of the ICD was used to extract hospitalized cases of CRF. Our research data was collected from the first pages of medical records at three tertiary first-class hospitals in Beijing. In 2016, the main diagnosis code of N18 (ICD-10 CRF code) was identified for hospitalized patients, and a total of 1,819 hospitalized cases were included.

Under the guidance of relevant reports (25–29), we included the following variables from the medical records: input variables included sex, age, marital status, medical payment method, length of stay, the number of other diagnoses, major procedures, and the number of procedures, and the target variable was hospitalization expenses (Table 1). The main procedure classification was adopted from the third volume of the American International Classification of Diseases Clinical Revision ICD-9-CM-3 (2011).

**Table 1:** Variables in the research.

**Variables**	**Variable assignment**	**Type of variable**
Gender	1 = male, 2 = female	Nominal variables
Age	—	Continuous variables
Marital status	1 = unmarried, 2 = married, 3 = other	Nominal variables
Medical payment method	1 = Medical insurance, 2 = full public expense, 3 = full self-pay, 4 = other	Nominal variables
Hospitalization frequency	—	Continuous variables
Number of other diagnoses	—	Continuous variables
Major procedure^*^	1 = 38.93; 2 = 38.95; 3 = 39.27; 4 = 39.42; 5 = 39.95; 6 = 54.93; 7 = 55.23; 8 = 55.69; 9 = 87.03; 10 = 87.41; 11 = 88.01; 12 = 88.72; 13 = 88.75; 14 = 88.76; 15 = 88.77; 16 = 93.96; 17 = null	Nominal variables
Number of procedures	—	Continuous variables
Length of hospital stay	—	Continuous variables
Hospitalized expense	—	Continuous variables

* *38.93, Venous catheterization; 38.95, venous catheterization for renal dialysis; 39.27, arteriovenostomy for renal dialysis; 39.42, revision of arteriovenous shunt for renal dialysis; 39.95, hemodialysis; 54.93, creation of cutaneoperitoneal fistuna; 55.23, closed (percutaneous) (needle) biopsy of kidney; 55.69, other kidney transplantation; 87.03, computerized axial tomography of head; 87.41, computerized axial tomography of thorax; 88.01, computerized axial tomography of abdomen; 88.72, diagnostic ultrasound of heart; 88.75, diagnostic ultrasound of urinary; 88.76, diagnostic ultrasound of abdomen and retroperitoneum; 88.77, diagnostic ultrasound of peripheral vascular system; 93.96, other oxygen enrichment*.

### Data Preprocessing

According to the research purpose and the meaning of each variable value, each variable was adjusted, and variables with a small sample size (<10) were deleted. Multiple variables in the major procedures category with fewer than 10 cases were deleted, including procedures that are not frequently performed during CRF patients' hospitalization. Finally, 1,635 valid hospitalized cases with no missing values constituted the data set for analysis.

Since hospitalization frequency, age, length of stay, the number of other diagnoses, the number of procedures, and hospitalization expenses are continuous variables, by calculating skewness and kurtosis, these variables were all found to follow a skewed distribution; therefore, the continuous variables were grouped, the sample frequency and composition ratio were used for descriptive statistics, and the median and quartile of the total medical expenses for each group were calculated. The same methods were used for sex, marital status, major procedures, and medical payment method.

IBM SPSS Statistics 23 downloaded from IBM official website was used for statistical analysis of the above data set.

### Random Forest Analysis

Due to the particularity of positive skewed distribution of medical expenditures, the variable types of latent factors included nominal variables and continuous variables, and the continuous data were also skewed. Related studies have used RF models for predictions (22), and other studies have shown that the RF method is a suitable ensemble learning algorithm and machine learning method with the advantages of no restrictions on variable conditions (30) and higher accuracy, sensitivity, and specificity than decision trees (31). In addition, RF can be used to predict continuous variables and obtain predictions without obvious deviations (32). Therefore, RF is a suitable prediction method for the data in this study.

### Least Absolute Shrinkage and Selection Operator

Least absolute shrinkage and selection operator penalty regression is another predictive model suitable for our research data. By constructing a penalty function, the coefficients of variables can be compressed to solve the problem of regression model overfitting. Least absolute shrinkage and selection operator is a regression technique for variable selection and regularization to enhance the prediction accuracy and interpretability of the statistical model that it produces. In LASSO, data values are shrunk toward a central point, and this algorithm aids in variable selection and parameter elimination. This type of regression is well-suited for models with high multicollinearity. Least absolute shrinkage and selection operator regression adds a penalty equal to the absolute value of the magnitude of coefficients, and some coefficients can become zero and are eventually eliminated from the model, resulting in variable elimination, and thus models with fewer coefficients (20, 33).

### Prediction Performance Evaluation

In this study, the mean absolute error (MAE) and root mean square error (RMSE) between the predicted value and the actual value were used to evaluate prediction performance. The coefficient of determination ***R***^**2**^ was used to reflect the regression fitting effect of the prediction model. The mean accuracy was used to assess the relative importance of variables (34).

The above algorithms were implemented using the LassoCV package and RandomForestRegressor package of sklearn in Python software.

## Results

### Sample Characteristics

Among the 1,635 hospitalized cases (see Table 2), males and females accounted for 58.6 and 41.4% of the sample, respectively; unmarried people accounted for 7.9%, married people accounted for 89.5%, and others accounted for 2.6% of the sample. Arteriovenostomy for renal dialysis (ICD-10 procedure code: 39.27) accounted for the largest proportion of major procedures at 24.3%, and other oxygen enrichment procedures (ICD-10 procedure code: 93.96) accounted for the smallest proportion at only 1.3%. In terms of medical payment methods, medical insurance accounted for the highest proportion at 71.3%, and fully public expenses accounted for the smallest proportion at 2.9%. A total of 41.9% of hospitalized patients were hospitalized for the first time, and the remaining 58.1% of hospitalized patients were hospitalized for the second time or more. Patients with a hospital stay shorter than or equal to 10 days accounted for 61.4% of the sample, and patients with a hospital stay > 21 days accounted for only 8.1% of the sample. Patients with no other diagnoses or one other diagnosis accounted for the smallest proportion of the sample at only 2.5%, while the proportion of patients with the five other diagnoses accounted for the highest proportion of the sample at 18.8%. Patients who did not undergo procedures accounted for only 14.4% of the sample, and patients who underwent two or more procedures accounted for the highest proportion at 65.4%.

**Table 2:** Sample characteristics and analysis of differences in hospitalization expenses of chronic renal failure.

**Variables (Group)**	**No. of cases**	**Median**	**Lower quartile**	**Upper quartile**	**Test statistic**	***P***
	**(% Total *N* = 1,635)**		**(CNY)**	**(CNY)**	**(U/H)**	
**Gender**					**1.322**	**0.25**
Male	958 (58.6)	8,350	5,601	14,609		
Female	677 (41.4)	8,053	5,118	14,425		
**Marital status**					**5.322**	**0.07**
Unmarried	129 (7.9)	9,863	5,345	41,537		
Married	1,464 (89.5)	8,126	5,429	13,901		
Other	42 (2.6)	8,550	4,549	19,620		
**Major procedure**					**579.141**	<0.01^*^
Venous catheterization	26 (1.6)	11,808	6,137	19,764		
Venous catheterization for renal dialysis	186 (11.4)	11,537	7,163	18,503		
Arteriovenostomy for renal dialysis	398 (24.3)	7,444	5,007	11,528		
Revision of arteriovenous shunt for renal dialysis	25 (1.5)	8,448	3,975	14,542		
Hemodialysis	19 (1.2)	7,050	4,895	14,229		
Creation of cutaneoperitoneal fistuna	66 (4.0)	11,516	7,675	16,711		
Closed (percutaneous) (needle) biopsy of kidney	24 (1.5)	7,712	5,861	10,198		
Other kidney transplantation	157 (9.6)	71,483	57,862	86,866		
Computerized axial tomography of head	30 (1.8)	9,374	7,164	15,446		
Computerized axial tomography of thorax	47 (2.9)	10,305	6,886	16,066		
Computerized axial tomography of abdomen	36 (2.2)	8,398	6,375	13,620		
Diagnostic ultrasound of heart	138 (8.4)	6,711	5,077	8,649		
Diagnostic ultrasound of urinary	48 (2.9)	6,591	4,964	8,871		
Diagnostic ultrasound of abdomen and retroperitoneum	114 (7.0)	6,598	4,735	8,320		
Diagnostic ultrasound of peripheral vascular system	58 (3.5)	7,379	5,416	9,914		
Other oxygen enrichment	21 (1.3)	11,866	5,844	19,478		
No procedure	242 (14.8)	5,362	2,860	8,960		
**Medical payment method**					8.361	0.04^*^
Medical insurance	1,165 (71.3)	8,119	5,346	13,431		
Full public expense	48 (2.9)	8,203	5,609	16,726		
Full self-pay	288 (17.6)	9,059	5,529	22,650		
Other	134 (8.2)	8,131	5,337	20,752		
**Hospitalization frequency**					15.186	0.02^*^
1	685 (41.9)	8,643	5,621	16,890		
2	308 (18.8)	7,973	5,454	16,033		
3	176 (10.8)	8,220	5,118	13,072		
4	135 (8.3)	7,942	5,755	12,473		
5	77 (4.7)	8,122	5,327	13,228		
6	54 (3.3)	5,893	4,492	11,511		
≥7	200 (12.2)	8,084	5,269	12,505		
**Age**					4.069	0.25
≤ 18	18 (1.1)	9,746	5,702	17,590		
19–40	328 (20.1)	8,586	4,867	40,158		
41–65	695 (42.5)	8,023	5,112	14,025		
≥66	594 (36.3)	8,318	5,748	13,110		
**Length of stay**					890.755	<0.01^*^
≤ 10	1,004 (61.4)	6,050	4,261	8,227		
11–15	363 (22.2)	12,468	9,062	17,228		
16–20	136 (8.3)	20,821	15,238	50,553		
≥21	132 (8.1)	72,926	39,114	89,635		
**Number of other diagnosis**					57.722	<0.01^*^
≤ 1	41 (2.5)	12,993	2,981	59,377		
2	95 (5.8)	11,076	4,219	63,538		
3	165 (10.1)	6,785	4,396	15,175		
4	199 (12.2)	6,529	3,936	10,728		
5	308 (18.8)	7,710	5,496	13,416		
6	193 (11.8)	7,843	5,031	12,133		
7	174 (10.6)	7,908	5,743	12,740		
8	127 (7.8)	8,648	5,772	14,348		
9	107 (6.5)	8,959	6,977	13,913		
10	226 (13.8)	11,115	7,365	16,710		
**Number of procedure**					223.974	<0.01^*^
0	235 (14.4)	5,336	2,870	8,959		
1	330 (20.2)	6,045	3,921	9,310		
≥2	1,070 (65.4)	10,028	6,471	17,649		

*CNY, China Yuan*.

* *The significance level is 0.05*.

### Analysis of Differences in Hospitalization Expenses for Chronic Renal Failure

With hospitalization expenses as the target variable and sex, marital status, major procedures, and medical payment method as characteristic variables, the Mann-Whitney U-test and Kruskal-Wallis H-test were performed. The results showed no statistically significant differences in hospitalization expenses with respect to sex (*p* > 0.05), marital status (*p* > 0.05), and age (*p* > 0.05), but major procedures (*p* < 0.001), medical payment method (*p* < 0.05), the number of hospitalizations (*p* < 0.05), the length of stay (*p* < 0.001), the number of other diagnoses (*p* < 0.001), and the number of procedures (*p* < 0.001) were associated with statistically significant differences in hospitalization expenses (Table 2). Further *post-hoc* testing of the pairwise comparison results was performed. Using the Bonferroni method, the α level was adjusted to analyze whether there are significant differences between the variables with significant differences in hospitalization expenses.

Table 3 shows the statistically significant results of the pairwise comparisons. According to the results of the pairwise comparison of major procedures, the hospitalization expenses of patients receiving other kidney transplantation procedures (median: 71,483, QR: 57,862–86,866) and all other groups of patients were significantly different. Statistical differences were found between the hospitalization expenses of patients in the no procedure group (median: 5,362, QR: 2,860–8,960) and those of patients in the venous catheterization (median: 11,808, QR: 6,137–19,764), venous catheterization for renal dialysis (median: 11,537, QR: 7,163–18,503), arteriovenostomy for renal dialysis (median: 7,444, QR: 5,007–11,528), Creation of cutaneoperitoneal fistuna (median: 11,516, QR: 7,675–16,711), other kidney transplantation (median: 71,483, QR: 57,862–86,866), axial computed tomography of the head (median: 9,374, QR: 7,164–15,446), axial computed tomography of the thorax (median: 10,305, QR: 6,886–16,066), axial computed tomography of the abdomen (median: 8,398, QR: 6,375–13,620), and other oxygen enrichment (median: 11,866, QR: 5,844–19,478) groups. Statistical differences were found between the hospitalization expenses of patients receiving venous catheterization for renal dialysis (median: 11,537, QR: 7,163–18,503) and those of patients receiving arteriovenostomy for renal dialysis (median: 7,444, QR: 5,007–11,528), diagnostic ultrasound of heart (median: 6,711, QR: 5,077–8,649), diagnostic ultrasound of the urinary (Median: 6,591, QR: 4,964–8,871), diagnostic ultrasound of the abdomen and retroperitoneum (median: 6,598, QR: 4,735–8,320), and diagnostic ultrasound of the peripheral vascular system (median: 7,379, QR: 5,416–9,914). Statistical differences were found between the hospitalization expenses of patients in the cutaneoperitoneal fistula group (median: 11,516, QR: 7,675–16,711) and those of patients in the arteriovenostomy for renal dialysis (median: 7,444, QR: 5,007–11,528) and diagnostic ultrasound of the heart groups (median: 6,711, QR: 5,077–8,649). Statistically significant differences in hospitalization expenses were observed for patients receiving ultrasound of the urinary bladder (median: 6,591, QR: 4,964–8,871) and diagnostic ultrasound of the abdomen and retroperitoneum (median: 6,598, QR: 4,735–8,320). Statistical differences were found between the hospitalization expenses of patients receiving axial computed tomography of the head (median: 9,374, QR: 7,164–15,446) and those of patients receiving diagnostic ultrasound of the abdomen and retroperitoneum (median: 6,598, QR: 4,735–8,320). Statistical differences were found between the hospitalization expenses of patients in the computerized axial tomography of the thorax group (median: 10,305, QR: 6,886–16,066) and those of patients in the diagnostic ultrasound of the heart group (median: 6,711, QR: 5,077–8,649) and diagnostic ultrasound of the abdomen and retroperitoneum group (median: 6,598, QR: 4,735–8,320). In general, patients who underwent kidney transplantation (median: 71,483, QR: 57,862–86,866) had the highest hospitalization expenses, and patients who did not undergo procedures (median: 5,362, QR: 2,860–8,960) had the lowest hospitalization expenses.

**Table 3:** Significance results of pairwise comparison.

**Variables (pairwise comparisons between groups)**	**Test statistic (χ^2^)**	***P***	**Adj *P***
**MAJOR PROCEDURE**			
No procedure vs. other kidney transplantation	1,022.617	<0.01	<0.01
Diagnostic ultrasound of abdomen and retroperitoneum vs. other kidney transplantation	940.165	<0.01	<0.01
Diagnostic ultrasound of urinary vs. other kidney transplantation	908.169	<0.01	<0.01
Diagnostic ultrasound of heart vs. other kidney transplantation	889.803	<0.01	<0.01
Diagnostic ultrasound of peripheral vascular system vs. other kidney transplantation	835.000	<0.01	<0.01
No procedure vs. creation of cutaneoperitoneal fistuna	478.819	<0.01	<0.01
No procedure vs. venous catheterization for renal dialysis	452.071	<0.01	<0.01
No procedure vs. venous catheterization	445.069	<0.01	<0.01
No procedure vs. computerized axial tomography of head	433.798	<0.01	<0.01
No procedure vs. other oxygen enrichment	428.269	<0.01	<0.01
No procedure vs. computerized axial tomography of thorax	418.861	<0.01	<0.01
Diagnostic ultrasound of abdomen and retroperitoneum vs. creation of cutaneoperitoneal fistuna	396.367	<0.01	<0.01
Diagnostic ultrasound of abdomen and retroperitoneum vs. venous catheterization for renal dialysis	369.619	<0.01	<0.01
Diagnostic ultrasound of urinary vs. creation of cutaneoperitoneal fistuna	364.371	<0.01	<0.01
Diagnostic ultrasound of abdomen and retroperitoneum vs. computerized axial tomography of head	351.346	<0.01	<0.01
Diagnostic ultrasound of heart vs. creation of cutaneoperitoneal fistuna	346.005	<0.01	<0.01
Diagnostic ultrasound of urinary vs. venous catheterization for renal dialysis	337.624	<0.01	<0.01
Diagnostic ultrasound of abdomen and retroperitoneum vs. computerized axial tomography of thorax	336.409	<0.01	<0.01
No procedure vs. computerized axial tomography of abdomen	327.031	<0.01	0.01
Diagnostic ultrasound of heart vs. venous catheterization for renal dialysis	319.258	<0.01	<0.01
Diagnostic ultrasound of heart vs. computerized axial tomography of thorax	286.047	<0.01	0.04
Diagnostic ultrasound of peripheral vascular system vs. venous catheterization for renal dialysis	264.454	<0.01	0.02
Arteriovenostomy for renal dialysis vs. venous catheterization for renal dialysis	262.794	<0.01	<0.01
No procedure vs. arteriovenostomy for renal dialysis	189.277	<0.01	<0.01
Arteriovenostomy for renal dialysis vs. creation of cutaneoperitoneal fistuna	−289.542	<0.01	<0.01
Revision of arteriovenous shunt for renal dialysis vs. other kidney transplantation	−804.186	<0.01	<0.01
Closed (percutaneous) (needle) biopsy of kidney vs. other kidney transplantation	−807.044	<0.01	<0.01
Arteriovenostomy for renal dialysis vs. other kidney transplantation	−833.340	<0.01	<0.01
Hemodialysis vs. other kidney transplantation	−786.375	<0.01	<0.01
Computerized axial tomography of abdomen vs. other kidney transplantation	695.586	<0.01	<0.01
Computerized axial tomography of thorax vs. other kidney transplantation	603.756	<0.01	<0.01
Other oxygen enrichment vs. other kidney transplantation	594.348	<0.01	<0.01
Computerized axial tomography of head vs. other kidney transplantation	588.819	<0.01	<0.01
Venous catheterization vs. other kidney transplantation	−577.548	<0.01	<0.01
Venous catheterization for renal dialysis vs. other kidney transplantation	−570.546	<0.01	<0.01
Creation of cutaneoperitoneal fistuna vs. other kidney transplantation	−543.798	<0.01	<0.01
**MEDICAL PAYMENT METHOD**			
Medical insurance vs. full self-pay	−85.799	0.01	0.03
**HOSPITALIZATION FREQUENCY**			
6 vs. 1	217.457	<0.01	0.02
**LENGTH OF STAY**			
≤ 10 vs. 11–15	−540.129	<0.01	<0.01
≤ 10 vs. 16–20	−784.929	<0.01	<0.01
≤ 10 vs. ≥21	−960.299	<0.01	<0.01
11–15 vs. 16–20	−244.800	<0.01	<0.01
11–15 vs. ≥21	−420.169	<0.01	<0.01
16–20 vs. ≥21	−175.369	<0.01	0.01
**NUMBER OF OTHER DIAGNOSIS**			
4 vs. 8	−177.181	<0.01	0.04
4 vs. 9	−224.115	<0.01	<0.01
4 vs. 2	258.628	<0.01	<0.01
4 vs. 10	−290.421	<0.01	<0.01
3 vs. 10	−221.126	<0.01	<0.01
6 vs. 10	−201.079	<0.01	<0.01
5 vs. 10	−167.541	<0.01	<0.01
7 vs. 10	−157.444	<0.01	0.04
**NUMBER OF PROCEDURE**			
0 vs. ≥2	−417.254	<0.01	<0.01
1 vs. ≥2	−324.505	<0.01	<0.01

*Each row tests the null hypothesis that the Sample 1 and Sample 2 distributions are the same. Asymptotic significances (two-sided tests) are displayed. The significance level is 0.05*.

From the perspective of medical payment methods, fully self-pay patients (median: 9,058, QR: 5,529–22,650) had higher hospitalization expenses than those who pay for medical insurance (median: 8,119, QR: 5,346–13,431). From the results of the pairwise comparison of the number of hospitalizations, only the first hospitalization (median: 8,643, QR: 5,621–16,890) and the sixth hospitalization (median: 5,893, QR: 4,492–11,511) showed significant differences in hospitalization expenses.

From the results of the pairwise comparison of length of stay, significant differences were found between the hospitalization expenses of patients in each length of stay group: ≤ 10 days (median: 6,050, QR: 4,261–8,227), 11–15 days (median: 12,468, QR: 9,062–17,228), 16–20 days (median: 20,821, QR: 15,238–50,553), and ≥21 days (median: 72,926, QR: 39,114–89,635), indicating that a longer length of stay corresponds to greater hospitalization expenses.

Judging from the pairwise comparison of the number of other diagnoses between the groups, statistically significant differences in hospitalization costs were found between patients with four other diagnoses (median: 6,529, QR: 3,936–10,728) and patients with two (median: 11,076, QR: 4,219–63,538), eight (median: 8,648, QR: 5,772–14,348), nine (median: 8,959, QR: 6,977–13,913), and 10 (median: 11,115, QR: 7,365–16,710) other diagnoses. Statistically significant differences in hospitalization costs were identified between patients with 10 (median: 11,115, QR: 7,365–16,710) other diagnoses and patients with three (median: 6,785, QR: 4,396–15,175), five (median: 7,710, QR: 5,496–13,416), six (median: 7,843, QR: 5,031–12,133), and seven (median: 7,908, QR: 5,743–12,740) other diagnoses.

In terms of the number of procedures, statistically significant differences in hospitalization expenses were found between patients undergoing two procedures (median: 10,028, QR: 6,471–17,649) and patients undergoing no procedures (median: 5,336, QR: 2,870–8,959) or one procedure (median: 6,045, QR: 3,921–9,310), showing that hospitalization expenses are higher for patients undergoing two or more procedures.

### Model Construction and Parameter Tuning

**Figure 1:**
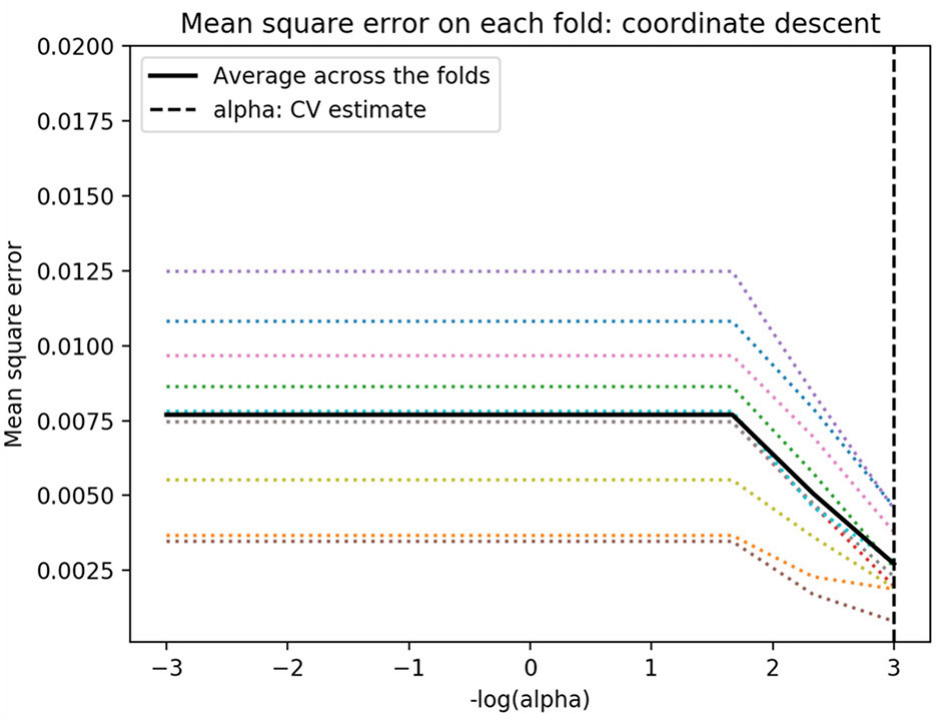
Relationship between Lambda and MSE.

Due to the small number of feature variables selected in this study and based on clinical experience, each variable had analytical value, and we therefore selected sex, age, marital status, medical payment method, hospitalization frequency, the number of other diagnoses, major procedures, the number of procedures, and hospitalization frequency as input variables and hospitalization expenses as the output variable. To reduce the influence of the unit difference between different variables, the linear conversion function *y* = (*x* – MinValue)/(MaxValue – MinValue) was used to normalize the variables. The study used 10-fold cross-validation to divide the entire sample into 10 equally sized subsamples. Among the 10 subsamples, one was retained as the verification data set of the test model, and the remaining nine were used as the training data set. After the cross-validation process was repeated 10 times, 10 results were generated (35), and the average value was taken as the performance metric. In this paper, the mean absolute error (MAE) was selected as the evaluation index, and the best Lambda value was obtained through cross-validation. The relationship between the model MSE and Lambda is shown in Figure 1. As shown in Figure 1, when the best penalty factor Lambda = 10^−3^, the MSE is the smallest, and the LASSO regression model has the highest accuracy. The multicollinearity problem can be solved by reducing the parameter Lambda. After performing cross-validation 10 times, the parameter n_estimator was trained in the RF model, and when it changed from 1 to 100 and n_estimator = 75, the ***R***^**2**^-value of the model was the largest.

### Evaluation and Comparison of Two Prediction Models

#### Performance Comparison

**Figure 2:**
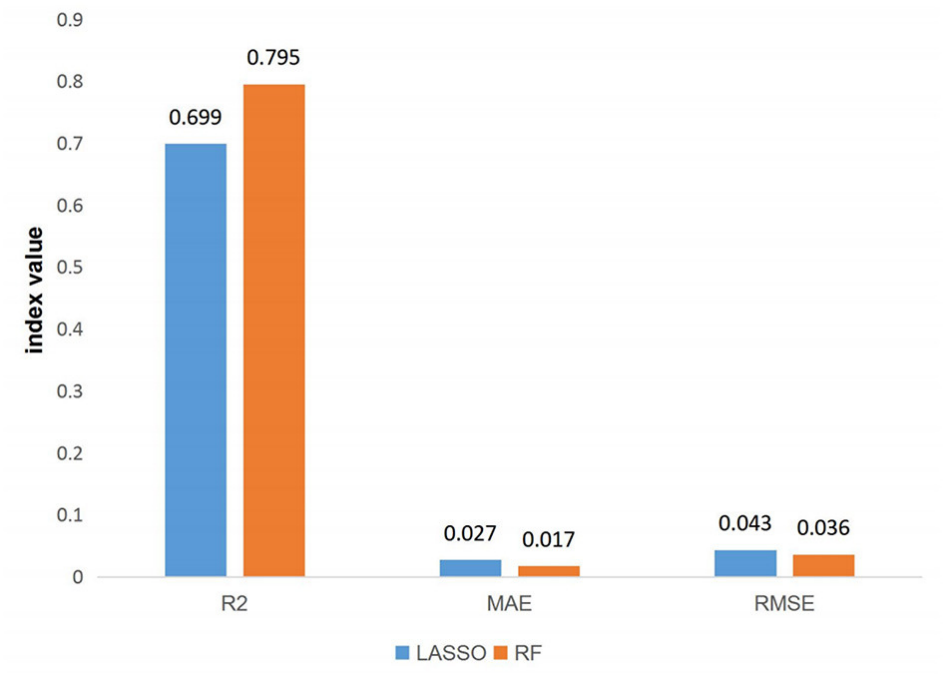
Comparison of model prediction performance.

Comparing the prediction performance of the RF prediction model and the LASSO regression model, in terms of the determination coefficient ***R***^**2**^, the ***R***^**2**^ of the LASSO regression model was 0.6992, and the ***R***^**2**^ of the RF regression model was 0.7946. The fitting effect of the RF prediction model was better than that of the LASSO regression model. The MAE and RMSE of the LASSO regression model were 0.0268 and 0.043, respectively, and the MAE and RMSE of the RF prediction model were 0.0171 and 0.0355, respectively. The prediction accuracy of the RF prediction model was better than that of the LASSO regression model (Table 4). The results were also shown in Figure 2.

**Table 4:** Comparison of model prediction performance.

**Model**	***R*^**2**^**	**MAE**	**RMSE**
LASSO	0.699	0.027	0.043
RF	0.795	0.017	0.036

#### Variable Selection Comparison

In the RF model, all input variables had a certain weight. The length of stay had the highest weight (0.730), followed by major procedures (0.089), and the variable with the lowest weight was marital status (0.004). In the LASSO model, only five variables had weights. The length of stay had the highest weight (0.604), followed by the number of other diagnoses (0.018) and the medical payment method (0.018). Four variables had a weight of zero, namely, major procedures, hospitalization frequency, the number of procedures, and marital status (Table 5).

**Table 5:** The related importance of variables.

**Variable**	**The variable weight of RF**	**The variable weight of Lasso**
Length of stay	0.730	0.605
Major procedure	0.089	0
Number of other diagnosis	0.067	−0.019
Age	0.050	−0.013
Medical payment method	0.031	0.018
Hospitalization frequency	0.015	0
Gender	0.007	−0.005
Number of procedure	0.006	0
Marital status	0.005	0

## Discussion

Our research results show that sex, age, and marital status produced no statistically significant differences in the hospitalization expenses of patients with CRF, but the results of Muñoz et al. (25) and Life et al. (36) both show a correlation between the age of patients with kidney disease and the cost of hospitalization, which may be related to sample selection in the study. The age range of the patients in the study sample is narrow at 44–73 years. With intensification of population aging, the medical and financial pressure caused by patients with CRF in various countries will inevitably increase, which illustrates the necessity of this study. In addition, future studies can expand the sample size, increase the age range of patients, and further explore the impact of age on the hospitalization expenses of patients with CRF.

Our research results also show that major procedures, medical payment methods, hospitalization frequency, the length of stay, the number of other diagnoses, and the number of procedures have a statistically significant impact on the hospitalization expenses of patients with CRF. The research of Zhao et al. (12) shows that medical insurance has no significant effect on medical expenses in China in contrast to the results of this study. However, the studies of Turenne et al. (14) and Hornberger et al. (15) both show that the medical payment method has an impact on end-stage renal dialysis methods and economic consumption. The study by Xiong et al. (37) also shows that the setting of different medical insurance policies has a certain impact on patients' medical expenses, which is consistent with our research results. Combining the important values of the predictive variables of the RF prediction model and the LASSO regression model and the results of related analyses, for patients with CRF, the main influencing factor for hospitalization expenses is the length of stay, which is consistent with the research results of Life et al. (36) and Arquivos de Neuro-Psiquiatria (38). The study by Wang et al. (22) showed that the length of stay and the main treatment methods are important factors affecting the hospitalization expenses of lung cancer patients, which is also consistent with our research results.

Our research results also show that with major procedures as the grouping variable, hospitalization expenses between the groups of CRF patients are not completely different. The median hospitalization expenses of the patients with kidney transplantation are the highest, and the median hospitalization expenses for patients without procedures are the lowest. No statistically significant difference in hospitalization expenses was found between HD and PD patients, showing that during a single hospitalization, these two treatment options are not the factors causing the difference in hospitalization expenses.

The length of stay was divided into groups and compared between groups. Significant differences in the median hospitalization expenses were found between the groups, and the hospitalization expenses increased with increasing hospitalization time. The cost of hospitalization for fully self-pay patients was higher than that of patients who pay for medical insurance. In terms of the number of procedures, the hospitalization expenses of CRF patients undergoing two or more procedures were higher than those of patients undergoing one or no procedures. According to the pairwise comparison between groups, hospitalization frequency and the number of other diagnoses had an impact on the hospitalization expenses of CRF patients, but the effect was not obvious.

Our research results also show that in the predictive model, major procedures had a relatively small impact on the hospitalization expenses of CRF patients. On the one hand, this finding may be related to the multicollinear relationship between major procedures and length of stay. With length of stay as the main influencing factor, the RF prediction model and LASSO regression model showed a smaller impact of major procedures on hospitalization expenses; on the other hand, according to major procedures performed, most cases had complications and accompanying symptoms.

Therefore, in addition to examinations and treatments for CRF such as kidney biopsy, HD, PD, and related medical procedures, many patients were examined and treated for other diseases, such as ultrasound examinations and oxygen therapy, indicating that patients with CRF undergo many examinations and treatments during hospitalization, which increases treatment expenses. Moreover, in addition to kidney transplant group, the hospitalization expenses of CRF patients in the medical group and non-operating room surgery group, did not significantly differ. At the same time, patients with CRF suffer from a variety of diseases, and their physical condition is poor. Therefore, the hospital stay will be relatively long, and medical resource consumption will increase accordingly, which may also be the reason why the length of stay had a greater impact on the hospitalization expenses of patients with CRF.

The evaluation and comparison results of the RF prediction model and the LASSO regression model show that the regression fitting and accuracy of the RF prediction model are superior to those of the LASSO regression model, and the LASSO regression model is more suitable for feature screening (33). In the RF prediction model, all input variables had a certain contribution to the model, but in the LASSO regression model, only five variables had a certain contribution, while the other four variables were not important to the model. The length of stay contributed the most to the two prediction models. Future research can also explore objective factors affecting the length of stay of CRF patients, such as age, complications, and comorbidities, to determine the appropriate length of hospitalization for individual patients and prevent inadequate hospitalization, which can affect clinical efficacy and prognosis. At the same time, hospitalization time can be effectively controlled, medical efficiency can be improved, and medical resources can be effectively allocated.

Based on the analysis results of the influencing factors of hospitalization expenses, we also believe that unlike patients submitted to short-term hospitalization for surgical procedures, most patients with CRF suffer from complications and comorbidities, resulting in diverse conditions, long hospital stays, and different hospitalization measures. The clinical process is highly heterogeneous, and the length of hospitalization also significantly differs due to individual differences. The two payment methods currently implemented in China, DRGs and DIPs, are mainly applicable to acute hospitalized cases (39) and are not suitable for patients with CRF. The CRF hospitalization expense prediction model based on the RF algorithm constructed in this study can be used to guide determination of the hospitalization expense reimbursement standard for individual patients with CRF, which can also be applied to the prediction and reimbursement of hospitalization expenses for other chronic and complex diseases.

Our research has some limitations. First, our study included only the first page of data from the medical records of three tertiary first-class hospitals in 2016, while other studies have a longer time span. Second, due to limited conditions, the sample data used in our research are not sufficiently comprehensive; future studies can further expand the sample size and sample scope and conduct more in-depth research. Finally, the dependent variables included in this study are limited, and future studies may consider including more dependent variables to explore the construction of predictive models with better performance.

## Conclusions

Our research shows that for inpatients with CRF in general hospitals, hospitalization expenses are affected by many factors such as length of stay, other diagnoses, medical payment methods, procedures, and the number of hospitalizations and that the degree of influence of each factor is also different, with length of stay being the most influential factor. The performance of the hospitalization expense prediction model constructed by the RF algorithm is better than that of the LASSO regression model. Using the RF prediction model to predict the hospitalization expenses of individual CRF patients is reasonable and convenient. In addition, the model represents an individualized and precise hospitalization cost compensation and control plan that health administration and medical security departments can consider implementing in the future.

## Data Availability Statement

The raw data supporting the conclusions of this article will be made available by the authors, without undue reservation.

## Ethics Statement

The studies involving human participants were reviewed and approved by the institutional ethics committees of the Third Xiangya Hospital Central South University (No:2020-s343). Written informed consent to participate in this study was provided by the participants' legal guardian/next of kin.

## Author Contributions

AL and PD conceived and designed the work. PD, WC, and ZX performed substantial contributions to the acquisition and analysis of data for the work. PD, HC, and WO interpreted the data for the work. All authors have participated in drafting the work or revising it critically, have done a final approval of the version to be published and agreement to be accountable for all aspects of the work in ensuring that questions related to the accuracy or integrity of any part of the work are appropriately investigated and resolved.

## Conflict of Interest

The authors declare that the research was conducted in the absence of any commercial or financial relationships that could be construed as a potential conflict of interest.

## Abbreviations

CRF: chronic renal failure
RF: random forest
LASSO: least absolute shrinkage and selection operator
CHD: in-center hemodialysis
RRT: renal replacement therapy
HD: hemodialysis
PD: peritoneal dialysis
DRGs: diagnosis-related groupings
DIP: diagnosis intervention packet
ICD: international classification of diseases
RMSE: root-mean-square error
MAE: mean absolute error
QR: quartile range.
